# The complete mitochondrial genome of *Saccostrea malabonensis* (Ostreida: Ostreidae): characterization and phylogenetic position

**DOI:** 10.1080/23802359.2022.2139160

**Published:** 2022-11-11

**Authors:** Wendan Mu

**Affiliations:** Fourth Institute of Oceanography, Ministry of Natural Resources, Beihai, China

**Keywords:** *Saccostrea malabonensis*, mitochondrial genome, phylogenetic analysis

## Abstract

The taxonomy of the genus *Saccostrea* is very confused, however, there is relatively little molecular information available on *Saccostrea*. In this study, we determined and described for the first time the complete mitochondrial genome of *Saccostrea malabonensis*. The complete mitogenome of *S. malabonensis is* 16,204 bp in length, containing 12 protein-coding genes (lack of *atp8* gene), two rRNA genes, 23 tRNA genes. The overall nucleotide composition of *S. malabonensis* has an AT content of 61.94% (26.29% A, 15.71% C, 22.35% G, 35.65% T). Phylogenetic analyses showed that *S. malabonensis* is first clustered with *S. cucullata* then united with *Saccostrea kegaki.*

Oysters are bivalve mollusks widely distributed in world estuaries and oceans, performing important roles in mitigating turbidity and improving water quality (Powell et al. 2018). They are sessile and benthic filter-feeders playing important roles in estuary ecology (Ren et al. 2010). Species in the genus *Saccostrea* are well known for high plasticity in shell with different habitat conditions, which leads to taxonomic problems (Lam and Morton 2006; Sekino and Yamashita 2016). The complete mitochondrial genome can provide useful information for species identification and studying phylogenetic relationships (Lei et al. 2010; Ma et al. 2012). Here we first sequenced the complete mitochondrial genome of *Saccostrea malabonensis* Faustino, 1932, and examined the phylogenetic position of *S. malabonensis* within Ostreidae.

The specimens of *S. malabonensis* were collected from Beihai, Guangxi province (21°0′55.9″N, 109°6′31.5″E), and preserved in 95% ethanol. Voucher specimens are deposited in the Fourth Institute of Oceanography, Ministry of Natural Resources, and the deposited numbers are IDNW8 (Wendan Mu, muwendan@4io.org.cn). Total genomic DNA was extracted from the ethanol-preserved tissue using TIANGEN marine animal DNA kit (TIANGEN, China). The complete mitochondrial genome of *S. malabonensis* was sequenced by Illumina NovaSeq6000 platform with 350 bp paired-end. The reads were assembled using SPAdes (v3.14.1). ORFfinder (http://www.ncbi.nlm.nih.gov/projects/gorf/orfig.cgi) and BLAST (http://blast.ncbi.nlm.nih.gov/Blast.cgi) were used to identify protein encoding genes and rRNA genes. The tRNA genes were identified by the program MITOS webserver (Bernt et al. 2013) and tRNAscan-SE 1.21 (Lowe and Eddy 1997). Sequences of twelve-partitioned amino acid sequences of protein-coding genes (excluding *atp8*) were aligned by MAFFT v7.037b (Katoh and Standley 2013). Poorly aligned sites were objectively eliminated with Gblocks ver. 0.91b (Castresana 2000). The appropriate evolutionary models for each data partition were selected using ProtTest version 3.4 (Darriba et al. 2011). Phylogenetic reconstruction was performed using Maximum Likelihood (ML) and Neighbor-Joining (NJ) with MEGA 5.0 (Tamura et al. 2011). The assessments of node reliability in the ML and NJ analyses were done by using 1000 bootstrap replicates.

The complete mitogenome of *S. malabonensis* is 16204 bp in length, containing 12 protein-coding genes (lack of *atp8* gene), two rRNA genes and 23 tRNA genes on the same strand (GenBank accession: ON649706). Previous studies on oysters (Ren et al. 2010; Volatiana et al. 2016; Wu JH et al. 2019; Li et al. 2021) showed the missing *atp8* gene, which is also confirmed in the present study. In contrast to the typical metazoan mitogenome, the large subunit rRNA gene (*rrnL*) is split into two fragments that occurs only in Ostreidae. *S. malabonensis* has an AT content of 61.94% (26.29% A, 15.71% C, 22.35% G, 35.65% T). Phylogenetic trees were constructed using the concatenated set of 12 protein-coding gene amino acid sequences of *S. malabonensis* and 20 published complete mitogenomes of mollusca. *Aplysia californica* was used as outgroup. Phylogenetic analyses showed that *S. malabonensis* is first clustered with *S. cucullata* then united with *S.kegaki* (Figure 1). Our phylogenetic analysis supports the current established taxonomic framework among the three genera of *Crassostrea*, *Ostrea* and *Saccostrea* in Ostreidae, and the relationships have also been verified by other molecular phylogenetic studies (Volatiana et al. 2016; Wu JH et al. 2019; Li et al. 2021). In conclusion, we expect that the complete mitogenome of *S. malabonensis* will provide important genome information for molecular phylogenetic studies on Bivalvia.

**Figure 1.**
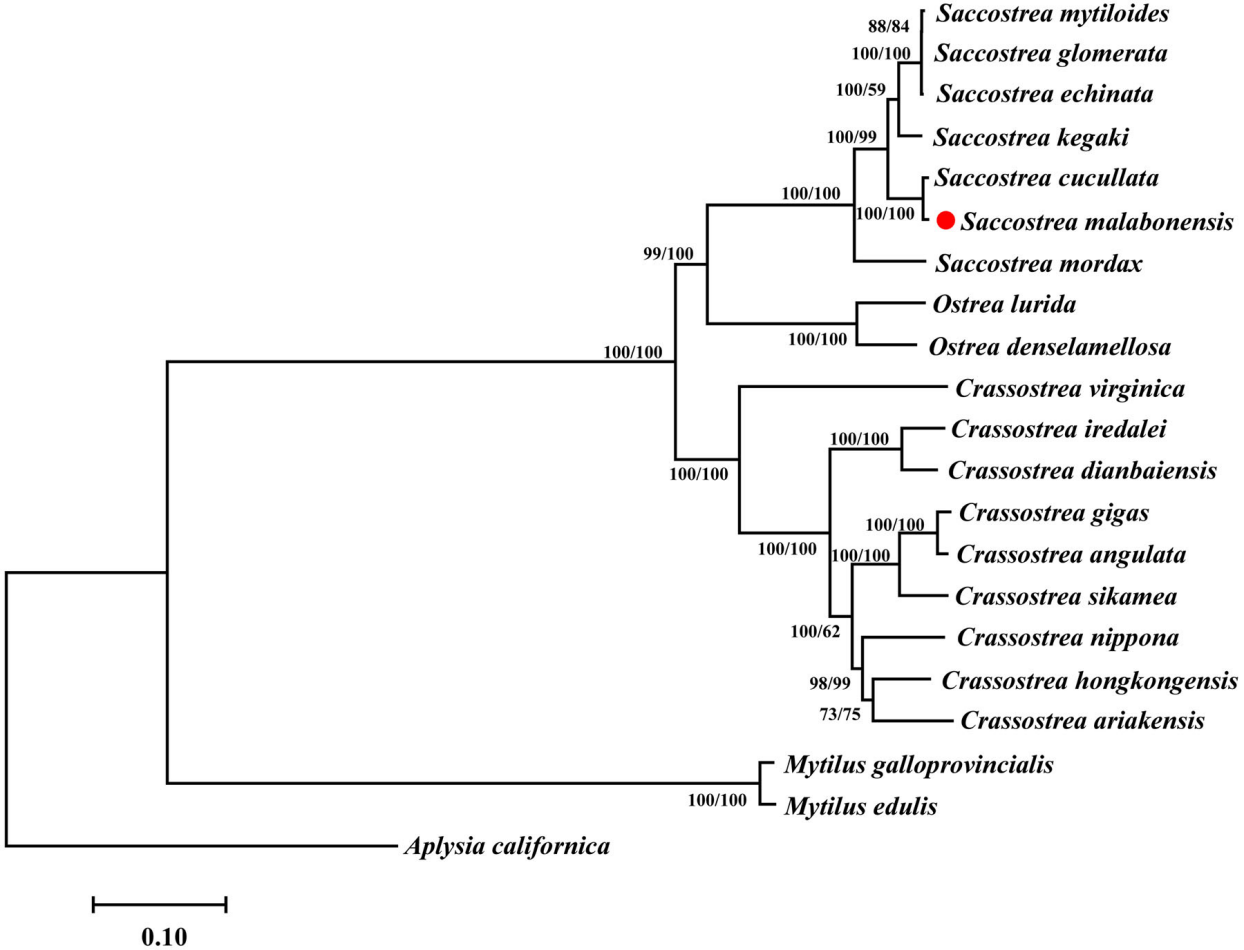
The branch length is determined with NJ analysis. NJ/ML bootstrap values are given for each branch. *Saccostrea mytiloides* NC_036479, *Saccostrea glomerata* NC_036483, *Saccostrea echinata* NC_036478, *Saccostrea kegaki* NC_030533, *Saccostrea cucullata* NC_027724 (Volatiana et al. 2016), *Saccostrea malabonensis* ON649706 (This study), *Saccostrea mordax* NC_013998, *Ostrea lurida* NC_022688 (Xiao et al. 2015), *Ostrea denselamellosa* NC_015231 (Yu and Li 2011), *Crassostrea virginica* NC_007175, *Crassostrea iredalei* NC_013997 (Wu X et al. 2010), *Crassostrea dianbaiensis* NC_018763 (Wu X et al. 2012), *Crassostrea gigas* NC_001276, *Crassostrea angulata* NC_012648 (Ren et al. 2010), *Crassostrea sikamea* NC_012649 (Ren et al. 2010), *Crassostrea nippona* NC_015248 (Yu and Li 2011), *Crassostrea hongkongensis* NC_011518 (Ren et al. 2009), *Crassostrea ariakensis* NC_012650, *Mytilus galloprovincialis* NC_006886 (Cao et al. 2004), *Mytilus edulis* NC_006161 (Boore et al. 2004), *Aplysia californica* NC_005827 (Knudsen et al. 2006).

## Data Availability

The data that support the findings of this study will be available in GenBank at https://www.ncbi.nlm.nih.gov/. The GenBank accession No. (reference number) is ON649706. The associated Bio-Sample, SRA, and BioProject numbers are SAMN28772344, SRR19514085, and PRJNA844164, respectively, and all of the accession numbers are activated.
